# Combined evaluation of audiology examination and self-reported symptoms in patients with hyperacusis

**DOI:** 10.1038/s41598-023-28570-8

**Published:** 2023-01-27

**Authors:** Yu Huang, Tao Xiang, Fan Jiang, Jing Ren, Tao Xu, Dan Lai

**Affiliations:** 1 Department of Otolaryngology Head and Neck Surgery, the Affiliated Hospital of Southwest Medical University, No. 25 TaiPing Street, Luzhou, 646000 Sichuan China; 2 Department of Otolaryngology Head and Neck Surgery, the Second Affiliated Hospital of Chongqing Medical University, Chongqing, China

## Abstract

To investigate the application of combined audiological examination and a self-reported symptoms survey in the evaluation of hyperacusis. Patients who visited the outpatient department of Otolaryngology Head and Neck Surgery and Otological medicine, Affiliated Hospital of Southwest Medical University, from January 2019 to March 2021 were divided into a hyperacusis group and a normal control group. We measured the loudness discomfort level (LDL) and hearing threshold (HT) of the subjects and investigated their self-reported symptoms. We compared the demographic characteristics, loudness discomfort level, and hearing threshold of the two groups and analyzed the self-reported symptoms and audiological characteristics of hyperacusis. We considered 87 subjects, comprising 40 patients with hyperacusis and 47 healthy individuals. Among the hyperacusis patients, bilateral disease was predominant. Among them, 33 were females, 23 had hearing loss, and 20 had tinnitus. Patients are mainly in the 21–60 age group. Patients with hyperacusis had low discomfort thresholds at all frequencies except 500 Hz (*P* ≤ 0.05, mean LDL decreased by 6.14–1.37 dB HL for all frequencies). The incidences of feeling upset, pain, and anxiety or fear were 95%, 65%, and 82.5%, respectively, in patients with hyperacusis. The severity of symptoms varies between patients with hyperacusis and healthy individuals. A combination of LDL measurements and self-reported symptom surveys allows for an accurate and comprehensive assessment of hyperacusis.

*Trial registration*: This study was retrospectively registered (TRN: ChiCTR2100047391) on June 13, 2021.

## Introduction

Hyperacusis, a disorder in loudness perception, lacks an internationally unified definition. Symptoms of hyperacusis mentioned in previous literature include hypersensitivity, annoyance, discomfort, unbearable fatigue, pain, abnormal lowered tolerance, and impairment in day-to-day activities due to environmental noise^1^. There are two commonly used descriptions of hyperacusis. First, it is thought to be related to sound sensitivity or tolerance, which has been described as hypersensitivity to common everyday sounds^2^. It can also be interpreted as sounds being perceived as moderately loud by those without hyperacusis and too loud by those with hyperacusis^3^. Hyperacusis has also been described as an abnormal uncomfortable response to sounds in daily life^4^, which often causes anxiety, fear, and pain, and can cause disruptions in sufferers’ daily lives as they attempt to avoid sounds^1^.

Tyler divided hyperacusis into four types according to the sufferers’ general perceptions and associated reactions. Loudness hyperacusis refers to acceptably loud sounds being unacceptably loud to sufferers. Annoyance hyperacusis sufferers experience negative emotions towards sound, such as getting distracted and tense. Fear hyperacusis is an avoidance behavior that occurs after hearing a sound. Pain hyperacusis involves feeling pain at moderately intense sounds^1^. However, it is difficult to distinguish the above types, because, in clinical practice, patients with hyperacusis often suffer from multiple symptoms.

Consequently, hyperacusis is considered a subjective symptom that is difficult to define and evaluate objectively^5^. At present, audiology measurements and questionnaires are the most popular hyperacusis assessment methods. The measurement of loudness discomfort level (LDL) is the most commonly used audiological method to assess hyperacusis. However, there is no uniform standard for its measurement. Some scholars have proposed that LDL lacks sensitivity and specificity and does not fully reflect the subjective symptoms of hyperacusis^6^. Several questionnaires assess hyperacusis from different aspects of auditory symptoms, such as the Geräuschüberempfindlichkeit (GÜF)^7^, the Hyperacusis Questionnaire (HQ)^5^, and the Multiple Activity Scale for Hyperacusis (MASH)^8^. HQ has been widely used in hyperacusis assessment, and its English, Italian, and Japanese versions are highly effective and reliable^5,9,10^. However, these questionnaires have not been validated in Chinese and cannot be easily applied in clinical practice in China.

Clinicians in China face severe challenges in clinical practice, such as a lack of time to interact with patients, which necessitates the use of a more convenient method to assess subjective symptoms. In particular, during the initial visit, it is difficult to fully understand the patient’s symptoms and make a correct diagnosis. At this point, the patient’s self-reported symptoms survey is particularly important. In addition to exploring the patient’s tolerance to pure tone loudness in a soundproof chamber, the self-reported symptom survey may better reflect the patient’s true tolerance to complex sounds in a non-soundproof environment, i.e., in real-life scenarios.

In this study, we measured subjects’ LDLs and designed a self-reported symptoms survey questionnaire for hyperacusis patients. Our survey was designed to explore the clinical characteristics of hyperacusis patients and to provide clinicians with a basis for taking medical histories and new ideas for assessing hyperacusis.

## Materials and methods

### Subjects

This study, which was approved by the Ethics Committee of the Affiliated Hospital of Southwest Medical University (registration number: KY2018005) and registered by the Chinese Clinical Trial Registry (registration number: ChiCTR2100047391), observed people in the outpatient department of Otolaryngology Head and Neck Surgery and Otological medicine, Affiliated Hospital of Southwest Medical University, from January 2019 to March 2021. This work is in accordance with the ethical principles stated in the Declaration of Helsinki. Data from individuals with an ability to understand audiological testing methods and the questionnaire content were included. People with other serious medical complications or mental illnesses that could not cooperate with the trial were excluded. During the study period, patients diagnosed by clinicians as hyperacusis were included in the hyperacusis group. And People without tinnitus and hyperacusis were selected as the normal control group. There was no difference in the composition of subjects in gender and age between the two groups. In our study, hyperacusis is defined as discomfort with sounds that the patient did not previously find uncomfortable or that are acceptable to most people in daily life^11^. All subjects provided informed consent and were tested at the Hearing Center, Affiliated Hospital of Southwest Medical University.

### Test protocol

Data collection involved two stages. First, participants were required to provide basic information, including name, sex, age, chief complaints, and disease course. They then underwent routine audiological examinations following the same protocol in a soundproof chamber including pure tone audiometry and loudness discomfort levels. The hearing thresholds (HTs) and the LDLs of the participants were measured in the order 1, 2, 4, 8, 0.25, and 0.5 kHz. Pure tone audiometry starts at a level at which the participant can hear and respond correctly at each frequency, reducing this level (in 10-dB HL steps) until the participant does not respond, and then increasing the level (in 5-dB HL steps) until the participant can respond. If the tester decreases and increases the hearing level five times and the participant responds more than three times at the same hearing level, this is recorded as the hearing threshold level. Hearing loss is considered the average hearing threshold (0.5, 1, 2, and 4 kHz) > 25 dB HL. The method of LDL is fully explained to the participant before the test, and the participant is told to react if he is uncomfortable and cannot accept the sound level. The measure of LDL starts from the hearing threshold level and gradually increases (in 5-dB HL steps). When the participant feels uncomfortable, the result is recorded as their LDL. If the person does not feel uncomfortable after reaching the maximum audiometer output, we define their LDL as the maximum output level (100, 120, 120, 120, 120, and 105 dB HL for 0.25, 0.5, 1, 2, 4, and 8 kHz, respectively) plus 5-dB HL^6^.

The second stage was a self-reported symptoms survey questionnaire consisting of eight items. In order to quickly understand the characteristics of hyperacusis patients during the consultation, the questionnaire items were limited and concise. It aimed to provide a comprehensive understanding of the important subjective symptom characteristics of patients with hyperacusis. To create this questionnaire, we tried to extract the experiences of hyperacusis patients from the previous literature, based on which seven items were selected for consideration. As the purpose of the questionnaire was to collect only the patients’ subjective symptoms, there was no dimensional division in the questionnaire design process. The contents of the questionnaire included: 1. expressed unacceptability of previously acceptable sounds; 2. could not tolerate sounds in daily life; 3. wanted to avoid exposure to sounds; 4. became upset about sounds in daily life; 5. became afraid of sounds in daily life; 6. experienced pain due to sounds in daily life; and 7. types of sounds. Five otolaryngology-head and neck surgeons were invited to review the questionnaire content and presentation. Given that hyperacusis and tinnitus are closely related and often coexist in clinical practice, the clinicians suggested adding an item to differentiate which disorder predominates in patients suffering from both conditions. Hyperacusis is a symptom of discomfort with external sounds, and when it is predominant, patients tend to prefer a quiet environment. In contrast, tinnitus is a self-induced ringing in the ear or head without a corresponding sound source, and when predominant, patients tend to prefer a slightly noisy environment. Upon completion of this phase, we generated an eight-item self-reported symptoms survey questionnaire.

The questionnaire included one binary question regarding the dominance of hyperacusis or tinnitus (item 1), one multiple-choice question about the type of sensitive sound (item 5), and six self-evaluation items (items 2, 3, 4, 6, 7, and 8). The answers in the above six items were given on a 4-point scale including no (scoring 0 points), yes, a little (scoring 1 point), yes, quite a lot (scoring 2 points), and yes, a lot (scoring 3 points). The sample size consisted of 87 subjects, 40 hyperacusis patients and 47 healthy individuals. After obtaining verbal consent, the subjects anonymously filled out the questionnaire. No respondents indicated difficulties in understanding the questionnaire, which was tested for reliability.

### Data analysis and statistical tests

We used SPSS 25.0 and Excel to build the database and analyze the data. Descriptive statistical analyses were reported, especially frequency, summation, mean, and standard deviation. T-test, Chi-Square test, and rank-sum test were used to compare demographic characteristics, questionnaire results, LDLs, and HTs between the two groups. Different measurement methods were reported by P-values, and the P-value for assessing statistical significance was an alpha of 0.05.

## Results

### General information

A total of 87 cases were considered in this study, of which 40 were hyperacusis patients and 47 were controls. There was no statistically significant difference between the two groups in terms of gender and age (*P* > 0.05). The basic characteristics of the subjects, including sex, age, hearing loss, tinnitus, affected side and the disease course, are summarized in Table 1. There were 63 ears with hyperacusis and 17 ears without hyperacusis in the hyperacusis patients. During the first visit, the complaints of hyperacusis patients were not all sensitivity to sound. Among the 40 people with hyperacusis, the incidences of chief complaints i.e., tinnitus, sensitivity to sound, self-reported hearing loss, earache, aural fullness, vertigo, and otorrhea, at the time of their first visit were ranked from highest to lowest (Table 2).

**Table 1:** Characteristics of subjects.

Characteristics		Hyperacusis N = 40(%)	Normal N = 47(%)	*P*-value
Sex	Female	33 (82.5)	37 (78.7)	0.658
	Male	7 (17.5)	10 (21.3)	
Age(years)	≤ 20	5 (12.5)	5 (10.6)	0.181
	21–40	16 (40)	24 (51.1)	
	41–60	15 (37.5)	18 (38.3)	
	61–80	4 (10)	0 (0)	
Disease course (months)	≤ 3	21 (52.5)		
	4–6	5 (12.5)		
	7–12	8 (20)		
	> 12	5 (12.5)		
	Uncertain	1 (2.5)		
Affected side	Right	5 (12.5)		
	Left	12 (30)		
	Bilateral	23 (57.5)		
Hearing loss	Yes	23 (57.5)		
	No	17 (42.5)		
Tinnitus	Yes	20 (50)		
	No	20 (50)

**Table 2:** Complaints of patients with hyperacusis at visit one.

Chief complaint	N = 40(%)
Sensitivity to sound	9 (22.5)
Tinnitus	15 (37.5)
Tinnitus and self-reported hearing loss	5 (12.5)
Self-reported hearing loss	2 (5)
Aural fullness	3 (7.5)
Earache	4 (10)
Vertigo	1 (2.5)
Otorrhea	1 (2.5)

### Audiological test results

**Figure 1:**
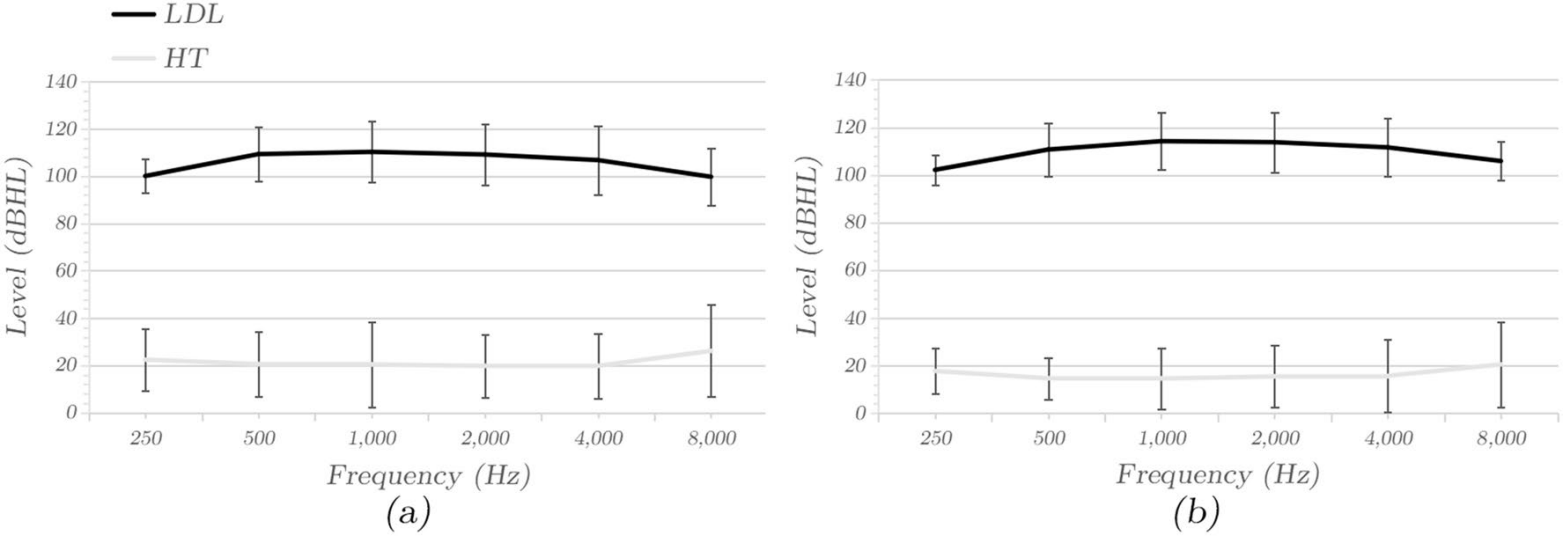
Hearing thresholds (HTs) and loudness discomfort levels (LDLs). (**a**) Average HTs (gray) and LDLs (black) of the hyperacusis group. Error bars denote ± 1 SD. (**b**) Average HTs (gray) and LDLs (black) of the normal group. Error bars denote ± 1 SD.

LDLs and HTs were measured in the hyperacusis and control groups, and the mean and standard deviation of each frequency were calculated. Interestingly, the mean LDLs at 0.25 kHz and 8 kHz were lower in patients and controls compared to the mean LDLs at other frequencies (Fig. 1). The hyperacusis group had a normal mean HTs (≤ 25 dB HL) in the 0.25 kHz to 4 kHz range and mild hearing loss at 8 kHz (Table 3). In contrast, the control group had normal mean HTs at all frequencies (Table 3). Compared to the controls, the mean LDL decreased by 6.14–1.37 dB HL for all frequencies in our hyperacusis group, but the mean HT increased by 5.83–4.01 dB HL for all frequencies (Table 3). The minimum decrease in mean LDL was observed for the 0.5 kHz frequency, while the maximum decrease in mean LDL was observed for the 8 kHz frequency. Patients with hyperacusis patients had lower LDLs compared to the control group at all frequencies except 500 Hz (*P* ≤ 0.05, Table 3). Hearing thresholds were higher in those with hyperacusis compared with the controls at all frequencies, and the differences were statistically significant (*P* < 0.05, Table 3).

**Table 3:** Comparison of the average LDLs and HTs for the hyperacusis and normal groups at different frequencies (Note. The number of ears included in each analysis is indicated by n. ).

		250 Hz	500 Hz	1 kHz	2 kHz	4 kHz	8 kHz	0.5-4 kHz
LDLs (SD)	Hyperacusis n = 63	100.08 (7.16)	109.37 (11.38)	110.32 (13.01)	109.21 (12.99)	106.83 (14.57)	99.76 (12.23)	108.92 (12.32)
	Normal n = 94	102.18 (6.37)	110.74 (11.07)	114.26 (12.00)	113.83 (12.69)	111.65 (12.10)	105.90 (8.10)	112.62 (11.30)
	*P*-value	0.003	0.315	0.034	0.009	0.031	< 0.001	0.05
HTs (SD)	Hyperacusis n = 80	22.41 (13.06)	20.51 (13.65)	20.37 (17.93)	19.75 (13.42)	19.75 (13.84)	26.14 (19.51)	19.78 (12.28)
	Normal n = 94	17.77 (9.49)	14.68 (8.70)	14.57 (12.78)	15.48 (13.06)	15.74 (15.28)	20.53 (17.85)	15.12 (11.52)
	*P*-value	0.004	< 0.001	0.004	0.003	0.004	0.022	< 0.001

### Self-reported symptom survey

The self-reported symptoms survey questionnaire had good readability, which was analyzed using items 2, 3, 4, 6, 7, and 8 with Cronbach alpha coefficients of 0.934. The results of the self-reported symptoms survey questionnaire are presented in Table 4. All (100%) of hyperacusis sufferers prefer to be in a quiet environment, even those with tinnitus (item 1). Items 2 and 3 were used as diagnostic criteria for hyperacusis. Patients with hyperacusis were all uncomfortable with sounds that had not previously been found to be uncomfortable or acceptable to most patients in daily life (item 2.3). Item 4.6.7.8 shows the effect and emotional response of hyperacusis on the patient. In item 4, 39 patients chose to avoid sound to avoid the discomfort caused by hyperacusis. The incidence of upset, pain, and anxiety or fear were 95%, 65% and 82.5% respectively in patients with hyperacusis (item 6.7.8). Symptoms vary between patients with hyperacusis, the most common being upset and avoidance behaviors. Item 5 categorized the sounds that the subjects felt discomfort with into any sound and specific sounds, and the results showed that 13 hyperacusis patients felt discomfort with any sound. 27 hyperacusis patients were only sensitive to specific sounds, such as a car horn, tapping of metal, rumbling of a machine, children crying, a mobile phone, and speakers and televisions (item 5). Hyperacusis patients who were uncomfortable with all sounds answered yes to item 2.3.4.6, and they answered yes to item 7.8 at a higher rate than hyperacusis patients who were uncomfortable with specific sounds. These 13 hyperacusis patients presented with seemingly more negative reactions than other hyperacusis patients.

**Table 4:** self-reported symptoms survey.

Item	Hyperacusis N = 40(%)	Normal N = 47(%)	*P*-value
1. Do you prefer quiet or slightly noisy environments?			
Noisy	0 (0)	0 (0)	
Quiet	40 (100)	47 (100)	
2. Are you uncomfortable with some everyday sounds you were comfortable with earlier?			< 0.001
No	2 (5)	47 (100)	
Yes, a little	20 (50)	0 (0)	
Yes, quite a lot	13 (32.5)	0 (0)	
Yes, a lot	5 (12.5)	0 (0)	
3. Do you find the everyday sounds in certain settings, such as streets, restaurants, KTV, wet markets, bars, bus stations, or concerts, are uncomfortably loud?			< 0.001
No	3 (7.5)	47 (100)	
Yes, a little	18 (45)	0 (0)	
Yes, quite a lot	9 (22.5)	0 (0)	
Yes, a lot	10 (25)	0 (0)	
4. Are there everyday sounds you wish you could avoid?			< 0.001
No	1 (2.5)	41 (87.2)	
Yes, a little	20 (50)	6 (12.8)	
Yes, quite a lot	12 (30)	0 (0)	
Yes, a lot	7 (17.5)	0 (0)	
5. Are you uncomfortable with the everyday sounds mentioned below?			0.02
All sounds	13 (32.5)	0 (0)	
Particular sounds			
Traffic/vehicle noise	17 (42.5)	9 (19.1)	
Metal hammering	17 (42.5)	9 (19.1)	
Children crying/noisy crowd	16 (40)	12 (25.5)	
Machines starting	16 (40)	0 (0)	
Mobile phones, speakers, or televisions	11 (27.5)	0 (0)	
Water flowing	2 (5)	0 (0)	
Drumming	2 (5)	0 (0)	
Blowing your nose	0 (0)	0 (0)	
6. Do you get upset when you hear noises or everyday sounds?			< 0.001
No	2 (5)	32 (68.1)	
Yes, a little	23 (57.5)	15 (31.9)	
Yes, quite a lot	11 (27.5)	0 (0)	
Yes, a lot	4 (10)	0 (0)	
7. Do you experience anxiety or fear when you hear noises or everyday sounds?			< 0.001
No	7 (17.5)	42 (89.4)	
Yes, a little	22 (55)	5 (10.6)	
Yes, quite a lot	8 (20)	0 (0)	
Yes, a lot	3 (7.5)	0 (0)	
8. Do you experience pain when you hear a noise or every day sound, such as a headache or earache?			< 0.001
No	14 (35)	44 (93.6)	
Yes, a little	11 (27.5)	3 (6.4)	
Yes, quite a lot	10 (25)	0 (0)	
Yes, a lot	5 (12.5)	0 (0)	

The control group did not experience discomfort with sounds that are acceptable to most people in their daily lives or that were previously acceptable. Some controls in Table 4 who preferred to stay in a quiet environment also felt uncomfortable with some high-frequency sounds, such as a car horn, metal tapping, and children crying. Even in the control group without hyperacusis, some subjects felt upset, anxious, fearful, painful and even wanted to avoid hearing the sound after hearing the high-frequency sound. However, the distribution of each symptom severity was different between the two groups.

## Discussion

In this study, HTs and LDLs values were measured in 87 subjects, and a self-reported symptoms survey questionnaire was collected. Similar to previous results^12–16^, our study shows that the prevalence of these conditions is higher in females than in males. Being female may be a risk factor for hyperacusis. In studies on the incidence of hyperacusis in the general population, the average age fluctuates between 35 and 57.8 years^14,16,17^, which is consistent with our study.

Most hyperacusis is bilateral^18^. 42.5% of patients with hyperacusis were unilateral in our study. The literature finds unilateral hyperacusis to be caused by a unilateral trigger such as acoustic shock and unilateral specific nerve injury^19^. Although the cause of hyperacusis is unknown for most patients, there are several diseases and syndromes associated with the condition, such as migraine, head injury, Williams syndrome, autism, myasthenia gravis, and middle cerebral aneurysm^1^. However, none of these diseases were found in our history survey of patients with unilateral hyperacusis. At present, the pathogenesis of hyperacusis has not been fully explained, but many scholars support abnormal auditory central gain^20,21^.

Our results show that subjects with hyperacusis often arrive at the clinic with tinnitus as the main complaint and that the prevalence of hearing loss and tinnitus is high among patients. The literature shows that the prevalence of hyperacusis in tinnitus patients can be 7.3–80%^15,22,23^, and the prevalence of tinnitus in hyperacusis can be 86%^24^. There is also a high probability of the simultaneous occurrence of hyperacusis and tinnitus. Some scholars believe that hyperacusis is a precursor of tinnitus^25^. Although hyperacusis and tinnitus are independent symptoms, they are subjective and often accompany each other, making it difficult for patients to distinguish between them. They can only be evaluated and differentiated through symptom surveys.

LDL measurement is one of the commonly used assessment methods for hyperacusis. Some studies have shown that LDLs below 100 dB HL might indicate hyperacusis^26^. Some experts report that an LDL at or above 80 dB HL is the normal range, while those lower than 80 dB HL at 0.5 and 2 kHz or lower than 75 dB HL at 4 kHz are abnormal^27^. In addition, Hashir Aazh proposed that the diagnostic criteria for hyperacusis are based on LDL: the lower average LDL of each ear at 0.25, 0.5, 1, 2, 4, and 8 kHz should be ≤ 77 dB HL^28^. There is no unified standard for the threshold value of LDL indicating hyperacusis. Previous studies also indicate that the sensitivity and specificity of LDL as a measurement indicator are poor^6^. Concurrently, the accurate acquisition of LDL is affected by subjective factors, such as the patient’s understanding of the test method and the level of cooperation between the examiner and the subject. In our data, the average LDL of each frequency of hyperacusis is > 90 dB HL, which may indicate hyperacusis. The true tolerance of patients in a non-soundproof room environment, or under complex sounds in real life, may not be easily restored by testing methods through the single-frequency pure tone. LDL, as an indicator of tolerance to pure tone loudness measured in a soundproof chamber, may not accurately reflect a patient’s true tolerance in daily life.

Hyperacusis is not only a problem of tolerance to sound intensity, but also involves emotional, social, and psychological effects. LDL is the maximum sound that can be tolerated, and it does not fully reflect the hyperacusis symptoms. The main content of the current popular questionnaire for hyperacusis assessment involves its impact on the attention, social activity, and emotions of sufferers, and the degree of annoyance in a particular living environment^5,7,8^. The self-reported symptoms survey questionnaire designed for this study captures the important hyperacusis clinical feature, and its main purpose is to collect the patient’s medical history rather than to assess hyperacusis severity. The questionnaire has a Cronbach alpha coefficient > 0.9, with high internal consistency and stability of the results. While completing the questionnaire, subjects self-assessed their symptoms to provide an understanding of the severity of each. The questionnaire provides a simple way to quickly identify tinnitus and hyperacusis and guidance to clinicians by collecting a comprehensive history in as short a time as possible. Tyler classified hyperacusis as loudness, annoyance, fear, and pain hyperacusis, and suggested that the emotional aspect of hyperacusis should be treated differently from its loudness aspect^1^. The loudness aspect of hyperacusis, i.e., the ability to tolerate sound, can be objectively measured by LDL. In contrast, the emotional aspects of hyperacusis can only be collected through questionnaires. The self-reported symptoms survey questionnaire designed in this study includes the subjective and the differential symptoms. Therefore, a combination of objective and subjective assessment methods provides a more comprehensive view of hyperacusis.

This study has several limitations. First, its limited sample size may influence the results, and the advantages of combined assessment should be demonstrated in a larger population. The newly proposed self-reported symptom surveys cannot evaluate the severity of hyperacusis, so we should further explore the Chinese version of a hyperacusis questionnaire suitable for medical environments in China.

## Conclusion

This study reports on the assessment of loudness discomfort levels and self-reported symptoms for hyperacusis. The measurement of LDL reflects sound tolerance and the questionnaire emphasizes the subjective symptoms of hyperacusis patients. Therefore, combining self-reported symptoms and LDL may provide a more comprehensive response to hyperacusis.

## Data Availability

All data generated or analyzed during this study are included within the article. The raw data are available in the Chinese Clinical Trial Registry. (TRN: ChiCTR2100047391).
